# Darkness stopping play? An update on cricket and mental health

**DOI:** 10.17159/2078-516X/2023/v35i1a15206

**Published:** 2023-06-05

**Authors:** T McCabe, R McCrea-Routray

**Affiliations:** 1 NHS Greater Glasgow and Clyde, University of Glasgow, Scotland; 2 Chief Medical Officer, Cricket Scotland, Edinburgh, Scotland

**Keywords:** cricket, franchise cricket, mental health, psychiatry, womens cricket

## Abstract

Mental health within elite cricket continues to be an area of focus for researchers and practitioners working within the game. Support structures for psychological issues within differing administrations and franchises vary. This may lead to inconsistent practice and levels of resource allocation. Elite level cricketers are exposed to stressors as a result of the congested international and domestic calendar, contract insecurity, injury and pressure to perform. Within the following commentary, the authors consider the existing medical literature, franchise and women-specific challenges and suggest ways to build on existing structures in order to optimise mental health within elite-level cricket.

Optimisation and awareness of mental health within cricketing populations has come into greater focus of late, in part due to campaigns and educational programmes provided by leading administrations and charities working within the game. ^[1]^ There has also been a societal shift and reduction in stigma with softening of attitudes toward mental health and illness following the COVID-19 pandemic in which traditional support structures were compromised and in-person socialisation was not possible. Given these factors, one could expect an increase in expressed psychological distress to be forthcoming. Do cricketing medical support teams have the ability to respond? We examine the cricketing landscape within the medical literature and suggest how resources may be harnessed in order to meet the need.

## The state of play

Thus far, high performance cricket and other elite sport have mostly relied upon estimates by way of self-report questionnaires to describe the prevalence of mental health symptoms within given cohorts of sportspersons. ^[1, 2]^ These studies suggest some of the most well-recognised mental health symptomology, such as poor sleep, anxiety, depression and adverse alcohol use to be at a similar level to that of the general population. Although diagnostic data exist within cricketing populations ^[2]^, it is unlikely that data pertaining to an enhanced epidemiological evidence base or studies examining the efficacy of specified interventions will be forthcoming in the short term. A recent editorial ^[3]^ highlighted the need for differentiation between natural fluctuations of mental state as a result of the psychological response to sporting experiences and diagnosable mental illness. In simplistic terms, cricketers may have features of heightened anxiety and low mood due to variations in form, psychological response to non-selection, and contract negotiations. In these circumstances, there may not necessarily be a need of a formal biopsychosocial formulation but rather a short period of support or informal intervention. Finding the ‘right level’ of support, at the ‘right time’, therefore can be challenging and may be dependent on resources.

## Franchise cricket

Outside of the international and test game, franchise cricket leagues are an established part of the competitive cricket calendar. The Indian Premier League is one of the most lucrative cricket leagues from a financial perspective, with other major competitions, such as Cricket Australia’s Big Bash and the English Cricket Boards T20 Blast, all vying for the recruitment of the world’s leading players. Additionally, the SA20 and UAE T20 cricket leagues started in 2023 and attracted worldwide interest. Competition congestion has led to some players and support staff choosing to ‘sub-specialise’ exclusively to the white ball game ahead of first-class or test cricket, with the primary driver moving towards financial gain and avoidance of burnout rather than a cricket skill-based decision. Some of the world’s leading cricketers have been prominent and open in the media with regards to taking time away from the game in order to recuperate as a result of mental stress. This is not routinely reported or disclosed within other sports. There is limited research available to describe any link between contract length and mental health although, Hendricks et al ^[1]^ suggests the optimal length of contract from a mental health perspective is that of two years.

With elite level players frequently representing different teams on different continents in relatively short periods of time, there can be little thought given to psychological rest. Players may be reliant on differing sources of support between these teams. Building and the implementation of a biopsychosocial model for an individual can take more than one sitting and is dependent on trust between the player and the practitioner, which takes time to cultivate. The optimal time to consider this may not be during an intense period of competition such as can be seen in franchise tournaments. Furthermore, ethical dilemmas exist in terms of communication between practitioners and a handover of care should one be required.

## Women’s cricket

Women’s cricket continues to flourish with a more prominent international calendar and rapidly evolving professionalisation. The Women’s Big Bash League (WBBL) in Australia, Women’s Premier League (WPL) in India and The Hundred in the UK currently lead the way in the women’s franchise game. Growing professionalisation brings differing challenges, including increased pressure on performance, negotiation of contracts along with an increased training and playing load outside of the International and domestic cricket calendar. The effect of this has yet to be described in the literature from a qualitative perspective. Sports medicine clinicians commonly address female athlete health specific aspects of care such as menstrual health, relative energy deficiency syndrome (RED-S) and its relation to performance and injury. ^[4]^ Within the mental health sphere, this should be similarly reflected. Women are reported to have higher levels of anxiety and reported disordered eating when compared to that of males and additionally, females within contact sports appear to take longer to recover from sports related traumatic brain injuries. ^[4]^ These factors suggest the need for differing focus when targeting prevalent symptoms and may require specialist expertise when allocating support resources.

## Getting on the front foot

Optimisation of mental health is a complex process, with each individual having unique experiences of psychological distress. Adaptability to provide holistic care for players and staff can be challenging to achieve from an organisational perspective but it is necessary with regard to the sport's rapidly evolving requirements. We know social networks, family and friends can have a major influence on the player’s mental state which is compromised when the player is away from their home environment for extended periods of time. ^[5]^ Elite teams now take this into account when considering scheduling. Governing bodies and emerging high-performance structures have an onus on them to have the appropriate support in place to meet the perceived increased need for psychological expertise for professional teams and players.

Traditionally, players have been reliant on a reactive model of care to respond to their mental health needs. There can be large variances in practice, help-seeking behaviours, levels of mental health literacy and ultimately, health outcomes within cricketing organisations. Similarly to injury of other areas of the body, identification of illness at the earliest possible stage is something which should be a primary aim. Purcell et al. ^[6]^ describe a framework for achieving this in which an ecological systems model for mental health performance is explained and discussed. In order to make a sustainable impact within this field and move beyond reactive care, there is a clear need for governing body investment and sharing of best practices.

## Conclusion

In conclusion, cricket has an opportunity to build on and expand the mental health support provided for players. Achieving this will require focus from all those within cricketing administrations, including franchise owners, directors of cricket, player representatives, as well as those with clinical insight. Sport and exercise physicians should aim to be in a position of leadership to work alongside specialist mental health experts in developing already established practices and delivering high-quality evidence-based interventions. Promoting player welfare, as well as influencing administrators of the game to prioritise mental health to achieve parity alongside that of the physical welfare should now be possible.
